# Progesterone prevents epithelial-mesenchymal transition of ovine amniotic epithelial cells and enhances their immunomodulatory properties

**DOI:** 10.1038/s41598-017-03908-1

**Published:** 2017-06-19

**Authors:** Angelo Canciello, Valentina Russo, Paolo Berardinelli, Nicola Bernabò, Aurelio Muttini, Mauro Mattioli, Barbara Barboni

**Affiliations:** 1 Faculty of Bioscience and Technology for Food, Agriculture and Environment, University of Teramo, Via Renato Balzarini 1, 64100 Teramo, Italy; 2 Istituto Zooprofilattico Sperimentale dell’Abruzzo e del Molise (IZSAM) “G. Caporale”, Campo Boario, 64100 Teramo, Italy

## Abstract

The *in vitro* expansion is detrimental to therapeutic applications of amniotic epithelial cells (AEC), an emerging source of fetal stem cells. This study provides molecular evidences of progesterone (P_4_) role in preventing epithelial-mesenchymal transition (EMT) in ovine AEC (oAEC). oAEC amplified under standard conditions spontaneously acquired mesenchymal properties through the up-regulation of EMT-transcription factors. P_4_ supplementation prevented phenotype shift by inhibiting the EMT-inducing mechanism such as the autocrine production of TGF-β and the activation of intracellular-related signaling. The effect of P_4_ still persisted for one passage after steroid removal from culture as well as steroid supplementation promptly reversed mesenchymal phenotype in oAEC which have experienced EMT during amplification. Furthermore, P_4_ promoted an acute up-regulation of pluripotent genes whereas enhanced basal and LPS-induced oAEC anti-inflammatory response with an increase in anti-inflammatory and a decrease in pro-inflammatory cytokines expression. Altogether, these results indicate that P_4_ supplementation is crucial to preserve epithelial phenotype and to enhance biological properties in expanded oAEC. Therefore, an innovative cultural approach is proposed in order to improve therapeutic potential of this promising source of epithelial stem cells.

## Introduction

Stem cells are object of intense studies and an emerging promise for cell-based therapy. Several researches demonstrate that amniotic membranes or amniotic-derived stem cells have great regenerative potential^1–4^. In particular, amniotic derived epithelial cells (AEC) have been largely studied^5^. In this regard, AEC display embryonic markers such as SSEA-3, SSEA-4, TRA-1-60 and TRA-1-81 and a subpopulation of cell express pluripotent genes (*Oct4*, *Sox2*, *Nanog* and *Tert*), in a highly conserved manner^2, 3, 6^. AEC differentiate into derivatives of all three germ layers despite they possess the phenotype of mature epithelial cells^2, 3, 7, 8^. Moreover, since placenta is the natural site where fetus-maternal immune tolerance is played, AEC display low immunogenicity^3, 9^. Indeed, several studies demonstrated that allo- and xenotransplantated AEC into immune competent organisms are well tolerated and survived in the host tissue without incurring in any rejection or tumorigenic derives^3, 5, 9, 10^. The long persistence of AEC in different host tissues is also due to their immunomodulatory and anti-inflammatory properties^7, 11–15^.

Although many efforts were made to standardize AEC cultural protocols in order to improve their therapeutic use, increasing evidences revealed that both the phenotype and the biological properties of these cells can be strongly affected by several factors related to cell origin (gestational stage of collection) or by *in vitro* manipulations^7, 16, 17^. Even though limited information are so far available on the effect of the gestational stage in human AEC (hAEC), since they are usually collected at labor^18^, strong evidences confirmed that epigenetic status, stemness and *in vitro* differentiation potential are all strictly related to the gestational stage in ovine model (oAEC)^7, 19^. However, the *in vitro* amplification has a negative influence on AEC phenotype, regenerative potential and immunomodulatory properties both in human and in animal models^7, 20^. In particular, hAEC undergo profound modification during *in vitro* amplification since they spontaneously experience epithelial-mesenchymal transition (EMT)^17, 21^.

The EMT is a trans-differentiation process whereby epithelial cells acquire a mesenchymal phenotype. EMT plays a crucial role in different processes such as embryogenesis, cancer progression and stem cells differentiation^22^. Extracellular signals such as TGF-β, Wnt and FGF modulates the EMT as well as its reverse process, mesenchymal-epithelial transition (MET)^23^. EMT is regulated by a family of transcription factors (EMT-TFs), including Snail, Twist and ZEB. They, in turn, are essential to promote the loss of epithelial proteins (E-Cadherin, Cytokeratin-8) and to up-regulate mesenchymal ones (Vimentin, *α*-SMA)^24, 25^. In particular, EMT dramatically alters the biological properties and the regenerative potential of AEC^7, 20^.

This phenotype and functional shift is supposed to be consequence of the inability to maintain in culture factors that physiologically modulate the amniotic cells homeostasis. Indeed, several factors disappeared after cell isolation such as low oxygen partial pressure and progesterone stimulation (P_4_)^26–28^. In this context, P_4_ may exert a key role in maintaining epithelial phenotype by actively inhibiting EMT as demonstrated in other cell models (ovarian, endometrial and metastatic breast cancer cells)^29–32^.

Starting from these premises, the present research has been designed to assess the effects of P_4_ supplementation during AEC amplification. The results confirmed the effective influence of P_4_ in preserving the oAEC epithelial phenotype. Indeed, P_4_ inhibited EMT pathway by preventing TGF-β secretion and by modulating EMT-related intracellular mechanisms. Noteworthy, P_4_-treated oAEC that preserved epithelial phenotype also displayed greater biological properties. In particular, oAEC acutely up-regulated stemness gene expression after P_4_ supplementation while enhanced their immunomodulatory properties, two functions, which are both crucial to impact on AEC regenerative potential.

## Results

### Cells in the epithelial layer of amniotic membranes express epithelial but not mesenchymal markers

**Figure 1:**
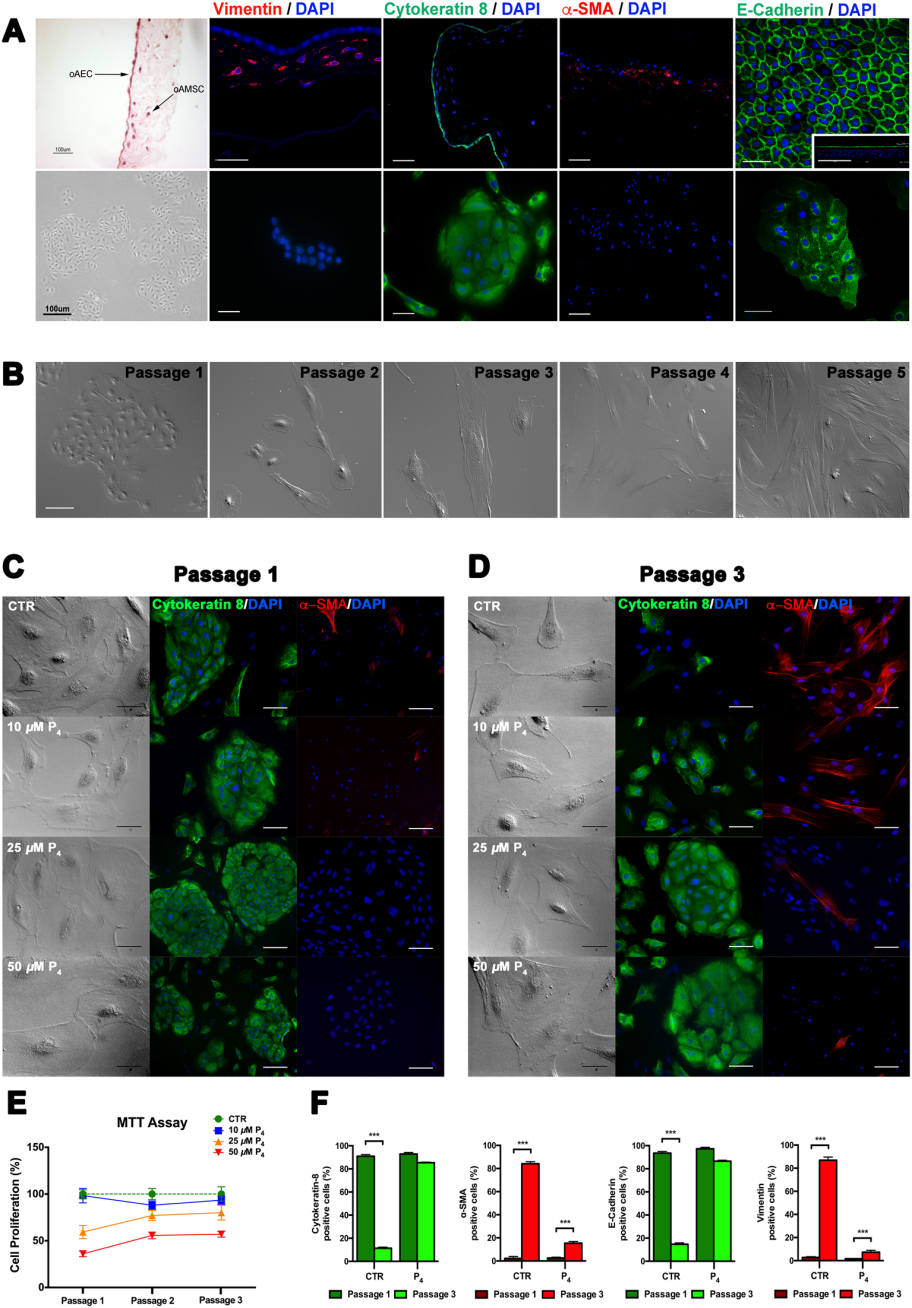
P_4_ prevents the *in vitro* EMT of oAEC. (**A**) Hematoxylin/eosin staining of amniotic membrane (AM) and phase contrast images of freshly-isolated oAEC show the same epithelial morphology. Scale bar: 100 *µ*m. Immunostaining for epithelial (Cytokeratin-8, E-Cadherin) and mesenchymal (*α*-SMA, Vimentin) markers in AM and freshly-isolated oAEC show the same expression pattern. Magnification 20x (AM Cytokeratin-8, *α*-SMA; oAEC *α*-SMA). Scale bar: 100 *µ*m Magnification 40x (AM Vimentin, E-Cadherin; oAEC Vimentin, Cytokeratyn-8, E-Cadherin). Scale bar: 50 *µ*m. (**B**) Morphology of oAEC in different passages. Scale bar: 50 μm. (**C**) Phase contrast images of CTR and P_4_-treated oAEC morphology. Scale bar: 25 *µ*m. Immunocytochemistry for Cytokeratin-8 and *α*-SMA, at passage 1. Scale bar: 100 *µ*m. (**D**) Phase contrast images of CTR and P_4_-treated oAEC morphology. Scale bar: 25 *µ*m. Immunocytochemistry for Cytokeratin-8 and *α*-SMA, at passage 3. Scale bar: 50 *µ*m. (**E**) Cell proliferation of CTR and P_4_-treated oAEC, during three passages. Data are expressed as percentage of proliferation ± SD, from *n* = 3 independent experiments performed in triplicate, with CTR set to 100%. (**F**) Fluorescence quantification of Cytokeratin-8, *α*-SMA, E-Cadherin and Vimentin protein expression in CTR and P_4_-treated oAEC, at passage 1 and 3. ***p < 0.001. Abbreviation: oAEC, ovine amniotic epithelial cells; oAMSC, ovine amniotic mesenchymal stem cells; CTR, control cell; P_4_, progesterone.

In order to determine the native state of oAECs, has been evaluated the expression of epithelial and mesenchymal markers in amniotic membrane (AM) and in freshly-isolated oAEC by immunostaining (Fig. 1A). Indeed, oAEC formed a well-compact monolayer in amniotic epithelial layer, whereas cells in amniotic mesoderm (oAMSC) were more dispersed and showed the typical mesenchymal shape. As showed in Fig. 1A, amniotic epithelial layer was positive for typical epithelial markers, Cytokeratin-8 and E-Cadherin, whereas amniotic mesenchymal layer was positive for mesenchymal markers, Vimentin and α-SMA. Nevertheless, freshly-isolated oAEC exhibited a typical epithelial shape in culture with a cobblestone-like morphology. Moreover, freshly-isolated oAEC were positive for Cytokeratin-8 and E-Cadherin as amniotic layer of AM with rare occurrence of Vimentin (2.8 ± 0.72%) and *α*-SMA (2.2 ± 1.70%) positive cells (Fig. 1A).

### oAEC lose their epithelial morphology during *in vitro* expansion

The typical oAEC epithelial morphology was progressively lost during *in vitro* amplification (Fig. 1B). Indeed, at the beginning of passage 3, oAEC dramatically lost their cobblestone-like morphology becoming more elongated. During subsequent passages, oAEC completely became fibroblastic-like cells (Fig. 1B). For this reason, passage 1 and passage 3 were selected as critical cultural points in order to evaluate oAEC changes in culture.

### P_4_ prevents the mesenchymal phenotype shift of oAEC amplified *in vitro*

oAEC cultured under standard cultural conditions (CTR) spontaneously turned into stromal-like cells (Fig. 1C and D). Only oAEC exposed to high concentrations of P_4_ (50, 25 and 10 *µ*M) maintained the original cobblestone-like morphology until passage 3 (Fig. 1C and D). At lower concentrations (from 5 to 0.01 *µ*M, data not shown) P_4_ lost any influence on cell morphology.

P_4_ supplementation (50, 25 and 10 *µ*M) had, in addition, a dose-dependent inhibitory influence on cell division as demonstrated by MTT assay results (Fig. 1E). The inhibition exerted by steroid supplementation on cell proliferation was dramatic at 50 *µ*M (approximately 60% lower than in CTR), slight at concentrations ranging from 25 to 10 *µ*M (approximately 40% and 2% lower than in CTR cells, respectively) whereas it did not display any effect at lower concentrations (5, 2.5 and 1 *µ*M, data not shown). The effects of P_4_ on cell proliferation persisted during cell amplification (Fig. 1E). Indeed, the proliferation index of 25 and 10 *µ*M P_4_-treated oAEC were respectively 20 and 6% lower than that recorded from CTR cells, at passage 3. Conversely, cells exposed to 50 *µ*M of P_4_ maintained a very low proliferation rate (approximately 40% lower than in CTR) (Fig. 1E).

High doses of P_4_ were, in parallel, able to preserve the epithelial phenotype of amplified oAEC. The positivity for Cytokeratin-8, which rapidly decreased during *in vitro* expansion under CTR conditions (from 90.2 ± 2.4% to 11.3 ± 1.1% at passage 1 and 3, respectively), was conversely retained in a dose-dependent manner in cells exposed to P_4_ (59.2 ± 4.2%, 85.3 ± 2.1% and 92.9 ± 2.7% positive cells exposed for three passages at 10 *µ*M, 25 *µ*M and 50 *µ*M P_4_, respectively; Fig. 1C and D). Simultaneously, P_4_ inhibited the appearance of mesenchymal markers. In fact, *α*-SMA positivity was proportionally reduced in amplified oAEC treated with 10 *µ*M, 25 *µ*M and 50 *µ*M of P_4_ (31.2 ± 5.2%, 15.1 ± 3.1%, 8.7 ± 2.5%, respectively; Fig. 1C and D). Similarly, 25 *μ*M P_4_ was able to maintain high expression of E-Cadherin (86.5 ± 0.86% of positive cells) with low expression of Vimentin (7.3 ± 1.66% of positive cells) in oAEC at passage 3. In contrast oAEC cultured under CTR condition progressively lost E-Cadherin expression (from 93.5 ± 1.48% to 14.7 ± 1.21% of positive cells) while increased Vimentin expression (from 2.8 ± 0.72% go 86.8 ± 2.75% of positive cells) (Fig. 1F).

Therefore, on the basis of dose-effect data, 25 *µ*M is the P_4_ concentration able to support over passages cell proliferation, in parallel with the preservation of epithelial phenotype. For this reason, this concentration has been then used for the following experiments.

### P_4_ interferes with EMT-related pathway

**Figure 2:**
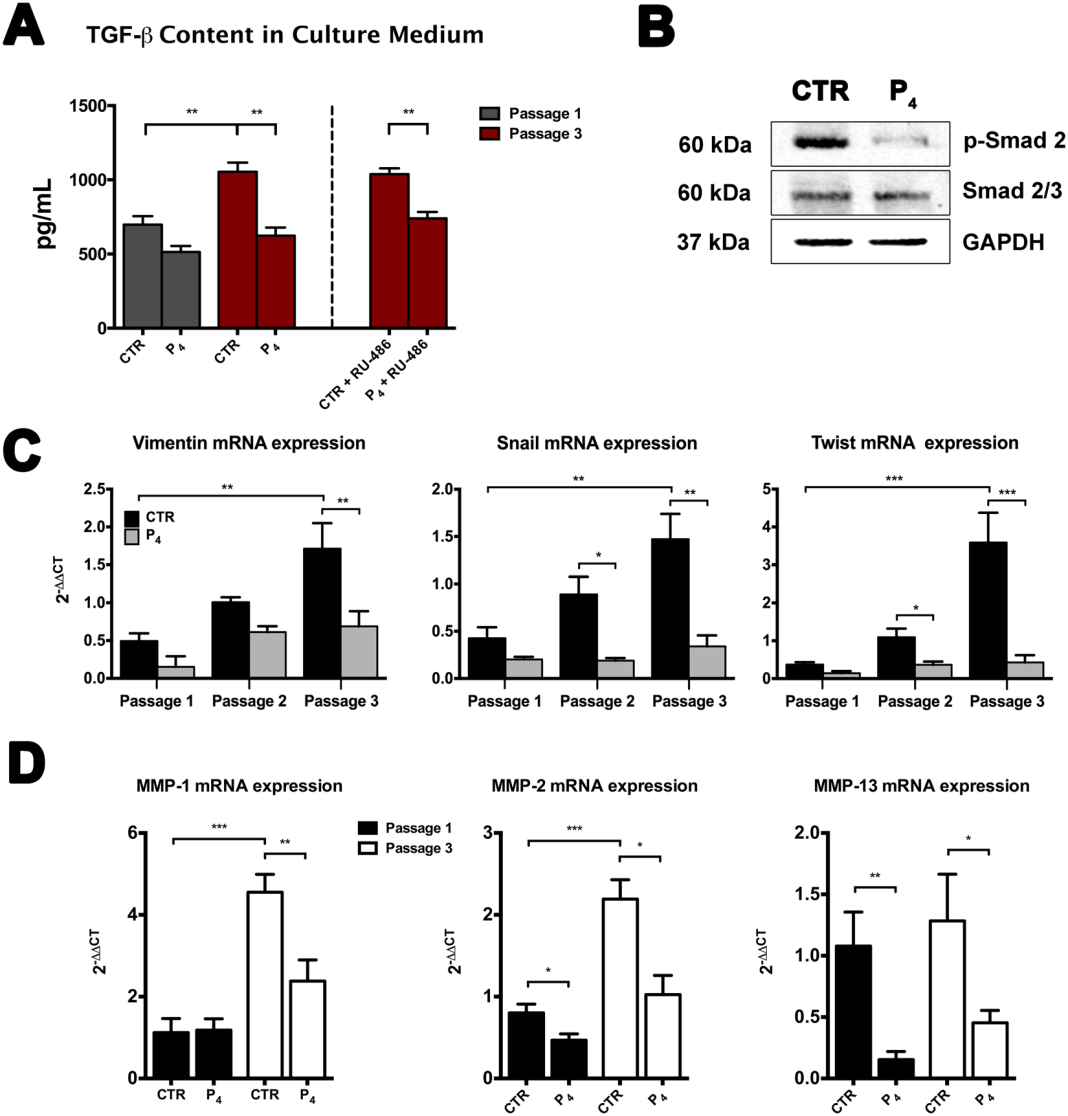
P_4_ interferes with TGF-β secretion. (**A**) TGF-β content in culture medium collected from CTR and P_4_-treated oAEC, at passage 1 and 3 with or without the addition of RU-486. Data are the mean ± SEM, from *n* = 4 independent experiments performed in triplicate. (**B**) Western blot analysis of p-Smad 2, Smad 2/3 and GAPDH in CTR and P_4_-treated oAEC, at passage 3. Full-length blots are presented in Supplementary Figure 1. (**C**) Real-time qPCR analysis of EMT-related genes (*Vimentin*, *Snail* and *Twist*) in CTR and 25 *µ*M P_4_-treated oAEC. The ΔΔ*Ct* values of passages 2 and 3 are calculated by normalizing the relative Δ*Ct* values against the Δ*Ct* values obtained from CTR and P_4_-treated oAEC at passage 1, respectively. (**D**) *MMP-1*, *MMP-2* and *MMP-13* mRNA expression in CTR and P_4_-treated oAEC at passage 1 and 3. Results are the mean ± SEM, from *n* = 4 independent experiments performed in triplicate. *p < 0.05, **p < 0.01 and ***p < 0.001. Abbreviation: CTR, control cell; P_4_, progesterone; RU-486, mifepristone; p-Smad 2, phosphorylated Smad 2; MMP, matrix metalloproteinase.

In order to define the mechanism of P_4_-mediated EMT inhibition, TGF-β content released in culture medium (CM) from CTR and P_4_-treated oAEC has been analyzed over passages (Fig. 2A).

CTR cells released high level of TGF-β in CM. Its concentration was slightly higher (697.6 ± 57.1 pg/mL) than P_4_-treated oAEC (513.6 ± 40.5 pg/mL), at passage 1 and became significant (1.4-fold higher than P_4_-treated oAEC, p < 0.01) at passage 3 (1003.0 ± 70.3 *vs* 677.6 ± 50.7 pg/mL, respectively). However, both CTR and P_4_-treated oAEC simultaneously exposed to selective nuclear receptor (PR) antagonist, mifepristone (RU-486), did not show any significant changes in TGF-β content released in CM, at passage 3 (Fig. 2A).

In order to determine wheatear, the amount of TGF-β released in CM from CTR and P_4_-treated oAEC was in its active form, has been evaluated the phosphorylation of Smad 2 protein (p-Smad), at passage 3 (Fig. 2B). Results indicated that P_4_ supplementation reduced the expression of p-Smad 2 in oAEC. Conversely, CTR that experienced EMT at passage 3 showed high level of p-Smad 2. In both cases, the expression of Smad 2/3 protein was not modulated (Fig. 2B).

Moreover, to better investigated the role of P_4_ in inhibition of EMT in oAEC, the expression of EMT-TFs (*Twist* and *Snail*) and *Vimentin*, has been evaluated over passages (Fig. 2C). Real-time qPCR analyses, demonstrated that P_4_ prevented the *Vimentin*, *Snail* and *Twist* expressions in amplified oAEC. The inhibitory effect of P_4_, already recorded at passage 1, persisted until passage 3. In detail, *Vimentin*, *Snail* and *Twist* expressions were approximately 2, 4 and 9-fold lower, respectively, in P_4_-treated oAEC than in cells incubated under CTR conditions, at passage 3 (Fig. 2C).

Furthermore, mRNA expression of matrix metalloproteinases (MMPs) has been evaluated in CTR and P_4_-treated oAEC, during *in vitro* culture. In particular, MMP-2 (p < 0.05) and MMP-13 (p < 0.01) showed higher levels of expression in CTR cells respect to P_4_-treated oAEC, at passage 1 (Fig. 2D). Conversely, even though an increase of all MPPs was found in both CTR and P_4_-treated cells at passage 3, the expression levels in CTR cells were always significant higher (p < 0.01 for MMP-1 and p < 0.05 for MMP-2 and MMP-13) respect to those recorded in P_4_-treated cells (Fig. 2D).

### RU-486 inhibits the effect of P_4_ on EMT

In order to evaluate wheatear P_4_ exerted its EMT inhibitory actions through PR, cells were exposed to RU-486.

**Figure 3:**
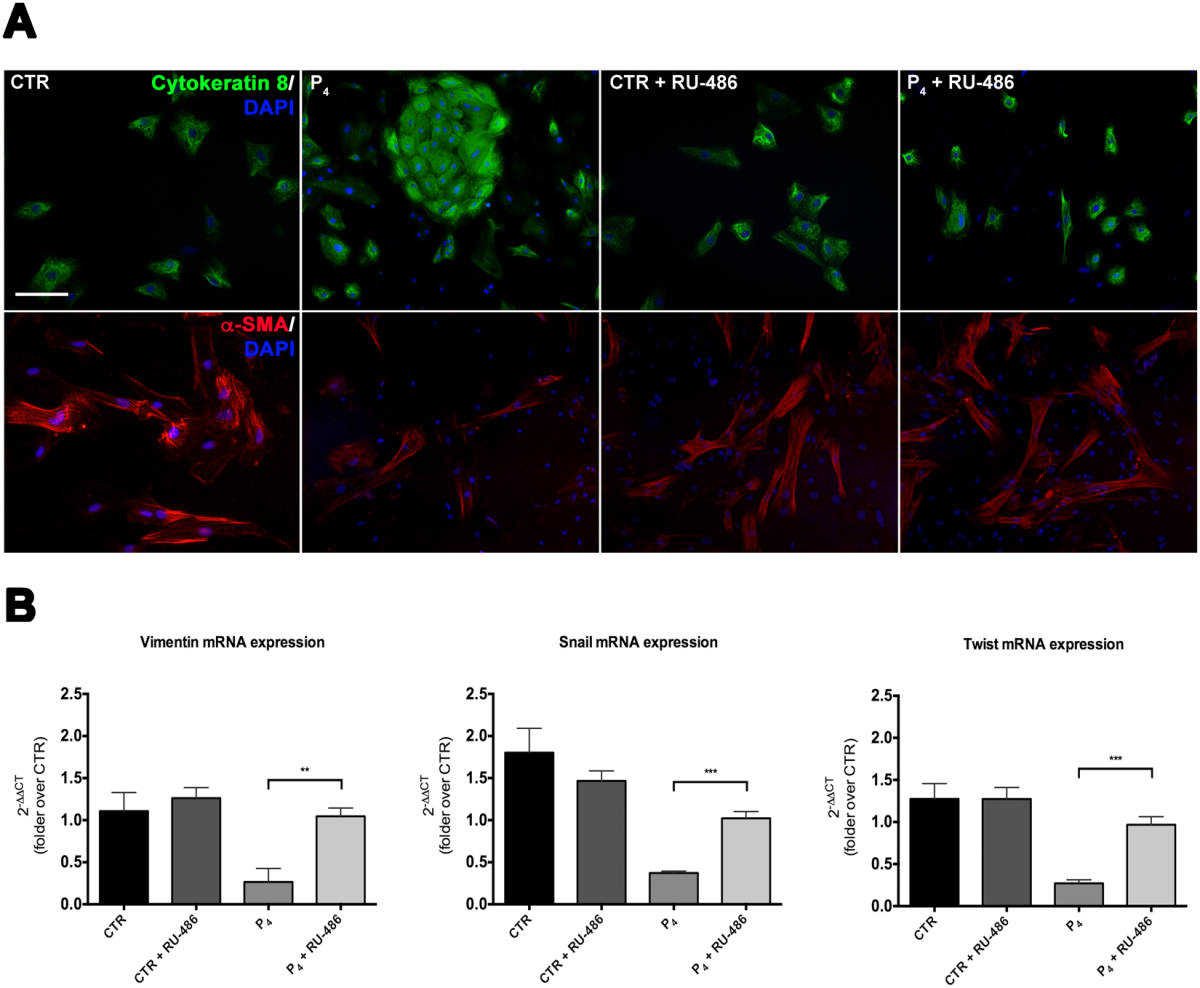
RU-486 (mifepristone) prevents the effect of P_4_ on EMT. (**A**) P_4_-treated oAEC exposed to RU-486 for 3 passages, progressively lost the Cytokeratine-8 positivity and increased the *α*-SMA expression as CTR cells. CTR exposed to RU-486 underwent EMT after 3 passages, and gained the expression of *α*-SMA. Scale bar: 50 *µ*m. (**B**) Real-time qPCR analysis of EMT-related genes *Vimentin*, *Snail* and *Twist*. Data are the mean ± SEM, from *n* = 3 independent experiments performed in triplicate. *p < 0.05, **p < 0.01. Abbreviation: CTR, control cell; P_4_, progesterone; RU-486, mifepristone.

The influence of P_4_ was completely reversed by the addition of RU-486 (Fig. 3A). Indeed, oAEC amplified with RU-486 in presence or absence of P_4_, progressively reduced the incidence of Cytokeratine-8 while increased *α*-SMA positive cells (Fig. 3A).

Moreover, PR also modulated the expression of EMT-related genes (Fig. 3B). In fact, oAEC simultaneously exposed to P_4_ and RU-486 display a significant higher expression of *Vimentin*, *Snail* and *Twist* than P_4_-treated cells (3.9, 2.7 and 3.7-folds, respectively: Fig. 3B).

### P_4_-treated oAEC does not undergo EMT upon steroid removal

**Figure 4:**
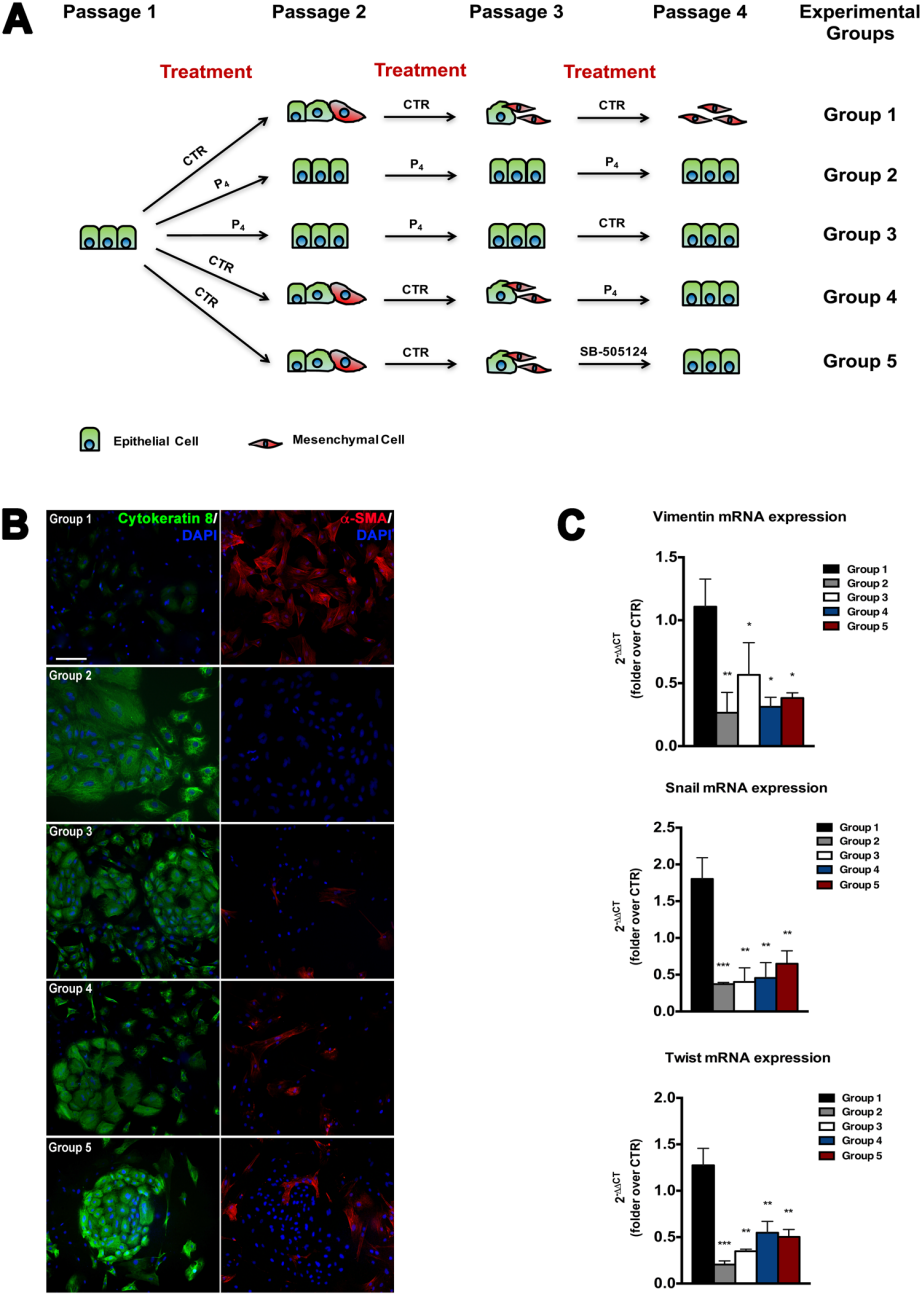
Effects of P_4_ withdrawal and P_4_ late exposure on oAEC phenotype and EMT-related gene expression. (**A**) Schematic representation of experimental design. Group 1 (CTR cells); Group 2 (P_4_-treated oAEC); Group 3 (P_4_ withdrawal from passage 3); Group 4 (CTR cells exposed to P_4_ from passage 3); Group 5 (CTR cells exposed to SB-505124 from passage 3). (**B**) Immunocytochemistry of Cytokeratin-8 and *α*-SMA in the different experimental groups. Scale bar: 100 *µ*m. (**C**) Real-Time qPCR analysis of EMT-related genes *Vimentin*, *Snail* and *Twist* in the same experimental groups. The ΔΔ*Ct* values of passages 4 are calculated by normalizing the relative Δ*Ct* values against the Δ*Ct* values obtained from CTR and P_4_-treated oAEC at passage 3, respectively. Data are the mean ± SEM, from *n* = 3 independent experiments performed in triplicate. *p < 0.05, **p < 0.01 and ***p < 0.001. Abbreviation: CTR, control cell; P_4_, progesterone.

In order to verify the persistence of P_4_ influence promoted *in vitro*, oAEC exposed to P_4_ for three passages were then cultured in a P_4_-free culture medium for one passage (Fig. 4B). In Fig. 4A, has been summarized the experimental design. CTR and P_4_-treated oAEC were referred as Group 1 and Group 2, respectively.

Interestingly, P_4_ removal for one passage (Group 3) did not affect the positivity for Cytokerartin-8 (80.6 ± 3.6% *vs* 85.3 ± 2.1% in Group 3 *vs* Group 2, respectively) and no significantly occurrence of *α*-SMA positive cells was recorded (19 ± 4.1% *vs* 15.1 ± 3.1% in in Group 3 *vs* Group 2, respectively) (Fig. 4B). By contrast, most of CTR cells (Group 1) became *α*-SMA positive, at passage 4 (83.5 ± 2.5% of positive cells).

These phenotype data were further confirmed by *Vimentin*, *Snail* and *Twist* expressions. In fact, Group 3 cells maintained unaltered their mRNA expression of EMT-related genes respect to Group 2 cells (Fig. 4C).

### P_4_ reverses EMT in amplified oAEC

The ability of P_4_ to reverse the EMT occurring *in vitro* has been considered. To this aim, oAEC amplified in CTR condition for three passages were then stimulated with the P_4_ for one passage. In Fig. 4A, has been summarized the experimental design. CTR and P_4_-treated oAEC were referred as Group 1 and Group 2, respectively.

Noteworthy, oAEC exposed to P_4_ (Group 4) significantly increased in one passage the incidence of Cytokeratin-8 positive cells (from 14.6 ± 3.1% to 66.3 ± 3.1%) by, simultaneously, reducing the expression of *α*-SMA (from 87.1 ± 2.2% to 21.3 ± 4.6%) (Fig. 4B).

In addition, the late administration of P_4_ (Group 4) promoted a prompt down-regulation of *Snail*, *Twist* and *Vimentin* (2.1, 2.4 and 3.5-fold decrease respect to CTR, respectively, p < 0.05 and p < 0.01) (Fig. 4C).

Finally, the late addition of a selective TGF-β signaling inhibitor (SB-505124) to oAEC cultured for three passages at CTR condition (Group 5) induced a rapid up-regulation of Cytokeratin-8 expression (Fig. 4B) with a concomitant down-regulation of EMT-related genes (Fig. 4C)

### P_4_ increases the expression of stemness genes on oAEC

The effect of P_4_ supplementation on stemness gene expression (*Oct4*, *Sox2* and *Nanog*) has been analyzed in oAEC during amplification.

**Figure 5:**
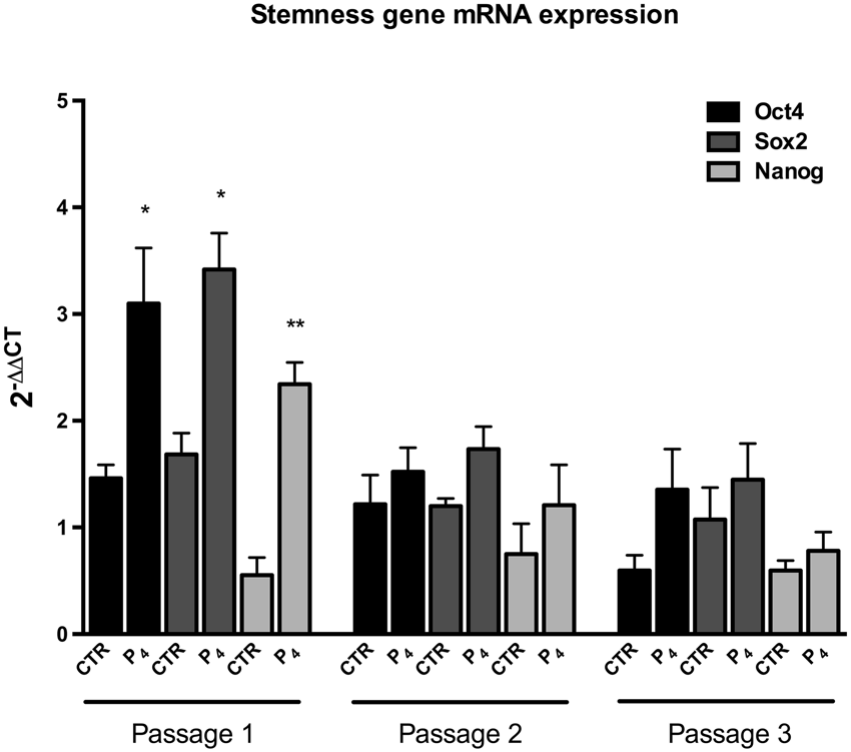
Effects of P_4_ on stemness gene expression. Real-time qPCR analysis of *Oct4*, *Sox2* and *Nanog* expression of CTR and P_4_-treated oAEC, during 3 passages. The ΔΔ*Ct* values of passages 2 and 3 are calculated by normalizing the relative Δ*Ct* values against the Δ*Ct* values obtained from CTR and P_4_-treated oAEC at passage 1, respectively. *p < 0.05 and **p < 0.01. Abbreviation: CTR, control cell; P_4_, progesterone. Abbreviation: CTR, control cell; P_4_, progesterone.

Intriguing, P_4_-treated oAEC significantly increased mRNA expression of *Oct4* (p < 0.05), *Sox2* (p < 0.05) and *Nanog* (p < 0.01) (2.1, 2.0 and 4.2-fold over CTR, respectively), at passage 1 (Fig. 5). However, the significant up-regulation induced by P_4_ was lost during next passages of amplification (Fig. 5).

### P_4_ influences the immunomodulatory responsively of oAEC

P_4_ influence to preserve, together with epithelial phenotype, also oAEC immunomodulatory activity has been investigated during *in vitro* amplification (at passage 1 and passage 3) by analyzing mRNA and protein secretion of anti- and pro-inflammatory cytokines.

**Figure 6:**
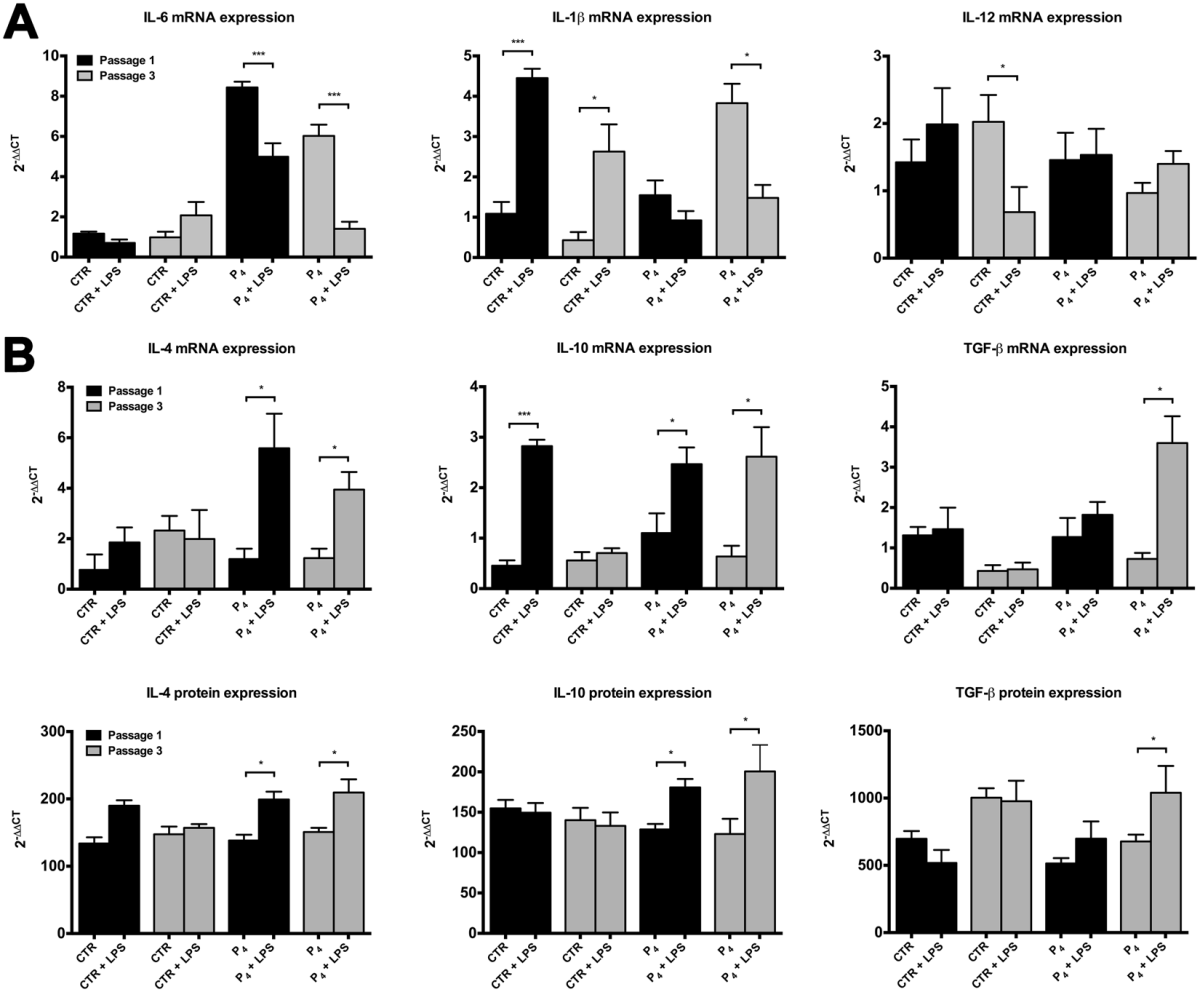
Effects of P_4_ on pro- and anti-inflammatory cytokines expression and release. (**A**) Comparison between basal and 24 h LPS-induced *IL-1β*, *IL-6* and *IL-12* mRNA expression in CTR and P_4_-treated oAEC, at passage 1 (black bars) and 3 (grey bars). (**B**) Comparison between basal and 24 h LPS-induced IL-10, IL-4 and TGF-β mRNA expression and protein release in CTR and P_4_-treated oAEC, at passage 1 (black bars) and 3 (grey bars). The ΔΔ*Ct* values of LPS-stimulated samples are calculated by normalizing against the Δ*Ct* values obtained from CTR and P_4_-treated samples without LPS, respectively. *p < 0.05 and ***p < 0.001. Abbreviation: CTR, control cell; P_4_, progesterone; LPS, lipopolysaccharide.

Basal mRNA expression of *IL-6*, *IL-1β* and *IL-12* was stable in CTR, during passages. In contrast, P_4_ supplementation modulated basal mRNA expression of pro-inflammatory cytokines. In detail, P_4_ induced a downregulation of *IL-6* (1.4-fold decrease from passage 1 to passage 3), an up-regulation of *IL-1β* (2.5-fold increase from passage 1 to passage 3) while *IL-12* remained stable during passages (Fig. 6A). After LPS stimulation, only CTR cells showed a significant up-regulation of pro-inflammatory *IL-1β*, both at passage 1 (p < 0.001) and passage 3 (p < 0.05) (Fig. 6A).

Basal expression of *IL-10*, *IL-4* and *TGF*-β was quite stable in culture regardless of cell treatments. Conversely, production of LPS-induced anti-inflammatory cytokines was strictly influenced by cell treatment (Fig. 6B).

At passage 1, LPS stimulated the expression of *IL-10* in both CTR and P_4_-treated oAEC (6.2 and 2.2-fold, respectively). The expression of *IL-4* increased (4.7-fold) exclusively in P_4_-treated cells, whereas mRNA levels of *TGF*-β were similar in CTR and P_4_-treated oAEC (Fig. 6B).

Noteworthy, the induction of cytokines expression upon LPS stimulation was retained, and in some cases increased, in P_4_-treated oAEC at passage 3. Indeed, cells exposed to P_4_ up-regulated 4.1, 3.2 and 4.9-folds *IL-10* (p < 0.05), *IL-4* (p < 0.05) and *TGF-*β (p < 0.05) expression, respectively. On the contrary, LPS stimulation was unable to stimulate over basal values the mRNA contents in anti-inflammatory cytokines of amplified CTR cells (Fig. 6B).

Finally, IL-10, IL-4 and TGF-β protein levels were recorded into cultural medium (CM) over passages (Fig. 6B). Basal IL-10 and IL-4 levels recorded in CM were stably secreted regardless of P_4_ stimulation whereas TGF-β significantly increased over passages only in CTR CM (Fig. 6B).

LPS stimulation exclusively increased IL-4 secretion in CTR, cells at passage 1 (p < 0.05; Fig. 6B). On the contrary, LPS stimulation positively modulated anti-inflammatory cytokines secretion in P_4_-treated oAEC (Fig. 6B). In particular, IL-10 content increased 1.4 and 1.6-fold, at passage 1 and 3 respectively (p < 0.05). IL-4 content increased 1.2 and 1.3-fold, at passage 1 and 3 respectively (p < 0.05) whereas TGF-β increased 1.5-fold exclusively at passage 3 (p < 0.05).

## Discussion

The present study demonstrates the role of P_4_ in preventing EMT and in preserving stemness and immunomodulatory properties of oAEC amplified *in vitro*. Indeed, freshly-isolated oAEC expressed high levels of epithelial (Cytokeratin-8 and E-Cadherin) and low levels of mesenchymal markers (α-SMA and Vimentin). However, after three cultural passages oAEC underwent EMT as confirmed by the decrease in Cytokeratin-8 and the increase in α-SMA positive cells. These two proteins have been selected as preferential EMT markers because of their rapid modulation during EMT and MET processes^33^.

The addition of P_4_ to oAEC culture strongly prevents this phenotypical and functional shift. The use of P_4_ has been proposed by taking into account its physiological role during pregnancy on fetus, uterus and fetal annexa^26–28^. Similarly, the amnion is under the influence of P_4_^27, 34, 35^. In fact, several evidences demonstrate its involvement in maintaining tight junctions amongst cells of epithelial layer^28^, in modulating receptivity^35, 36^ and regulating the secretion of inflammatory cytokines^37^. Although P_4_ physiologically regulates pregnancy at concentrations lower than those proposed *in vitro* ^26, 34^, when it is used on primary and immortalized cell lines P_4_ usually started to become effective at doses higher than 1 μM^30–32, 37, 38^. The dose-effect experiments carried out in the present research analogously show that P_4_ becomes inhibitory on cells proliferation at doses higher than 10 *µ*M whereas it prevents or reverses EMT even at higher concentrations (≥25 *µ*M). Noteworthy, high doses of P_4_ affects both chronically and acutely the phenotype and function of expanded oAEC. In particular, the effect of P_4_ in maintaining oAEC epithelial phenotype persists for one passage after hormone removal. Conversely, P_4_ supplementation immediately reverses mesenchymal phenotype of oAEC that have experienced EMT *in vitro*.

The present research also provides information on the signaling pathway involved in P_4_-preventing *in vitro* EMT. Firstly, P_4_ interferes in culture with TGF-β autocrine/paracrine signaling. TGF-β is physiologically secreted from amniotic cells *in vivo* ^39, 40^ and *in vitro* and is spontaneously accumulated during expansion, thus inducing EMT in human and oAEC as well as in others cancer cell lines^17, 22–24^.

This study demonstrates that oAEC actively release TGF-β during amplification and that its secretion is inhibited by the presence of P_4_. However, the treatment with progesterone nuclear receptor inhibitor (RU-486) does not reverse the effects of P_4_, thus indicating that the inhibition of TGF-β secretion is related to a different pathway. To better investigate the role of P_4_ in the inhibition of TGF-β intracellular signaling, the phosphorylation of Smad 2 protein has been studied. Results clearly indicate that P_4_ is able to prevent the phosphorylation of Smad 2 whereas CTR cells progressively increase the secretion of TGF-β and, in turn, the phosphorylation of Smad 2. Finally, the treatment with a potent TGF-β signaling inhibitor (SB-505124) induces the complete reversion of mesenchymal phenotype in oAEC that experienced EMT, thus confirming the involvement of TGF-β pathway in this process.

These findings are in accordance with results in the literature. To this regard, P_4_ signaling has a crucial role in inhibiting EMT in endometrial^30^ and breast cancer cells^31, 32^, thus reducing their proliferation and invasiveness. In particular, P_4_ realizes a potent anticancer effect in endometrial cells specifically through the inhibition of TGF-β pathway. More in details, P_4_ modulates TGF-β signaling in endometrial cancer cells at ligands, receptors, and Smads levels, thus inhibiting invasiveness as a consequence of an increase in E-cadherin and a decrease in Vimentin expression^41^.

Analogously, P_4_ repression of TGF-β/Smad 2 signaling in oAEC converges to a core regulatory network composed by EMT-TFs (*Snail* and *Twist*) and *Vimentin*. Indeed, both *Snail* and *Twist* act in concert to regulate the repression of epithelial genes and the up-regulation of mesenchymal ones, thus promoting EMT^42^. The down-regulation of both these hallmark genes represents a clear evidence of EMT inhibition^25^ in P_4_-treated oAEC. The stable lower expression of *Vimentin* recorded in P_4_-treated oAEC is an important confirmation that EMT is completely prevented^43, 44^. All these data seem to confirm that oAEC undergo EMT in culture throughout a TGF-β-mediated process actively inhibited by P_4_ and not as consequence of the prevalence of higher proliferating mesenchymal clones.

The EMT-inhibitory action of P_4_ is also substantiated by the ability of this hormone to revert mesenchymal phenotype in oAEC that experience EMT during amplification. In fact, P_4_ provides a prompt MET reprogramming and the recovery of the epithelial phenotype by a dramatic down-regulation of EMT-related genes, in oAEC. In literature, evidences that supported the involvement of P_4_ in MET program have been previously described exclusively in cancer cell lines^31, 32^.

Matrix metalloproteinases (MPPs) represent another hallmark of EMT process^45, 46^. During oAEC amplification the expression of *MMP-1*, *MMP-2* and *MMP-13* dramatic increase as consequence of *in vitro* culture and EMT process. Conversely, P_4_ supplementation strongly reduces the up-regulation of MMPs expression.

Some studies confirmed that P_4_ is able to inhibit Wnt/β-catenin signaling through PR in endometrial cancer model^30^. Indeed, P_4_ play a direct role in blocking the intracellular mechanism of EMT as confirmed by experiments conduct in presence of its nuclear receptor antagonist (RU-486), where oAEC undergo EMT likewise untreated cells. Moreover, the addition of RU-486 is sufficient to revert the effects of P_4_. In fact, RU-486 is able to prevent P_4_-mediated EMT inhibition allowing oAEC to undergo phenotypical shift by inducing an up-regulation of EMT-TFs.

Regardless of P_4_ EMT-inhibitory mechanisms, the present research highlights the role of P_4_ in preserving stemness and immunomodulatory properties of oAEC. Indeed, our results indicated that P_4_ is essential to enhance acutely pluripotency genes expression during the first passage as well as the ability to secrete high levels of anti-inflammatory cytokines after LPS stimulation. Both these cultural consequences may be positively correlated with AEC regenerative potential, since the former influences cell trans-differentiation^3, 5^ while latter affects cell engraftment and tissue regeneration mechanisms avoiding fibrotic derives^6, 7^.

It is well known that primary stem cells progressively lose plasticity and change differentiative abilities after extended *in vitro* culture^20, 47–49^. This negative consequence affects also AEC. Though plasticity of P_4_-treated cells has not been evaluated in the present research, the up-regulation of pluripotent genes (*Oct4*, *Sox2* and *Nanog*) has been demonstrated. This result seems to suggest an increase in stemness of P_4_-treated cells, a property that after *in vitro* amplification is greatly compromised as consequence of a pluripotency genes down-regulation^50^.

Although numerous advantages are derived from AEC use in tissue repair/regeneration and from their therapeutic potential in animal model of several diseases, AEC are found to make a more significant contribution in pre-clinical studies thanks to their anti-inflammatory and anti-fibrotic effects^16^. Indeed, it is well known that AEC secrete soluble factors with immunomodulatory, anti-inflammatory, angiogenic, anti-apoptotic and anti-oxidative properties^16^.

In this research, we demonstrated that oAEC amplification cause the loss of epithelial phenotype that, in turn, is responsible for the reduction of oAEC immunomodulatory properties. Conversely, P_4_ treatment is essential to preserve not only the native epithelial phenotype but in a long-term culture induces an increase in expression and release of the key anti-inflammatory cytokines, IL-10, IL-4 and TGF-β after LPS stimulation.

The role of P_4_ in modulating anti-inflammatory properties of hAEC has been extensively study^35, 38, 51^. To our knowledge, this is the first report to describe the effect of P_4_ in enhancing the expression and release of the key anti-inflammatory cytokines. The relevance of this finding is related to the direct inhibitory action of these molecules IL-10, IL-4 and TGF-β on innate and adaptive immune response^52^. These results also highlight the pleiotropic role played by TGF-β in oAEC. In fact, on one hand, the increase of basal TGF-β secretion is related to the induction of *in vitro* EMT whereas, on the other hand, an increase after LPS stimulation is related to immunosuppressive role of this cytokine during the inflammatory response^53^. In parallel, P_4_ supplementation induces a dramatic down-regulation of the keys pro-inflammatory cytokines (IL-1β, IL-6 and IL-12) expression upon LPS stimulation in both freshly-isolated and amplified oAEC. Therefore, P_4_ seems modulate the inflammatory response of oAEC toward a more immunomodulatory and immunosuppressive phenotype respect to oAEC that experienced EMT. Further experiments are needed to confirm the enhanced immunomodulatory properties also in *ex vivo* and *in vivo* models.

In conclusion, the *in vitro* amplification of AEC is necessary to obtain enough cell numbers for clinical applications but several issues are related to high cultural passages. Therefore, our cultural strategy could represent a critical standardization able to preserve *in vitro* the native biological and functional characteristics of these promising source of stem cells to improve *in vivo* their therapeutic and regenerative properties.

## Methods

### Ethic statement

None ethic statement is required for the present research since the cells were always collected from animals slaughtered for feed purposes.

### Reagents

Progesterone (4 pregnene-3,20-dione, P4), Lipopolysaccharide (LPS, from Escherichia coli 055:B5), Mifepristone (RU-486) and SB-505124 (S4696) were purchased from Sigma (St Louis, MO, USA).

### Cell isolation and culture

Sheep uteri were collected at a local abattoir from sheep of Appeninica breed at mid gestational stage determined on the basis of fetus dimension (25–30 cm length) and brought at approximately 25 °C to the laboratory in maximum 1 h, for further processing. Once the uterus wall was opened, amniotic membrane was mechanically peeled off from allantois and dissected in order to obtain pieces of approximately 3–5 cm. Membrane pieces, washed in Phosphate Buffered Saline (PBS), were incubated in 0.25% Trypsin-EDTA 200 mg/L at 37.5 °C for 20 min. The cell debris released during this digestion was discarded. The cell suspension obtained after an enzymatic digestion of further 30 min was filtered through a 40 *µ*m filter and the isolated cells were collected into a tube containing 10% Fetal Calf Serum (FCS, Lonza) in order to inactivate trypsin. Finally, the freshly-isolated oAEC were seeded in 50 mm dishes (Corning) at the final concentration of 3 × 10^3^ cells/cm^2^ in alpha minimum essential eagle medium (*α*-MEM, Gibco) supplemented with 20% FCS, 1% ultraglutamine (Lonza), 100 U/mL penicillin (Lonza), 100 *µ*g/mL streptomycin (Lonza) and, 2.5 *µ*g/mL amphotericin (Euroclone). The cells were incubated at 38.5 °C in 5% CO_2_ in the absence (CTR) or presence of different concentrations of P_4_ (0.01, 0.1, 1, 2.5, 5, 10, 25, 50 and 100 *µ*M). Medium as well P_4_ and P_4_ receptor antagonist (25 *µ*M Mifepristone, RU-486) were refreshed every two days. At 70–80% confluence, cells were dissociated by 0.05% Trypsin-EDTA and plated at the same concentration for subsequent passages.

Cell morphology was evaluated at medium refreshing when micrographs (40x magnifying objective) are captured by using Nikon inverted microscope ECLIPSE Ti connected with Nikon DS-Fi2 interfaced to a computer workstation, provided with NIS-Elements Advanced Research imaging software.

### Cytokeratin-8, α-SMA, E-Cadherin and Vimentin proteins detection

Amniotic Membrane (AM) and oAEC were evaluated for Cytokeratin-8, *α*-SMA, E-Cadherin and Vimentin by immunofluorescence analysis. To this aim, the cells were cultured in presence or absence of P_4_ on glass coverslips, whereas AM mechanically peeled off from allantois and were placed on glass coverslips.

Afterwards, oAEC and AM were fixed in 4% paraformaldehyde (10 min), permeabilized with 0.2% (v/v) Triton X-100 in PBS (10 min), incubated with 5% (w/v) BSA in PBS (1 h), each step at room temperature (RT), and then incubated with anti-Cytokeratin-8 (1:200) (Abcam), anti-*α*-SMA (1:200) (Abcam), anti-E-Cadherin (1:200) (ABIN1440031), anti-Vimentin (1:200) (M0725) antibodies, diluted in 1% (w/v) BSA/PBS, overnight at 4 °C. Finally, cells and AM were exposed to Cy3 or Alexa Fluor 488 conjugated anti-mouse secondary antibodies, diluted 1:200 in 1% (w/v) BSA/PBS (40 min) at RT. Nuclear counterstaining was obtained with 4′,6-diamidino-2-phenylindole (DAPI, Vectastain) used at the final dilution of 1:5000 in PBS. Coverslips were finally mounted with Fluoromount (Sigma Chemical Co.) and cell samples analysed using an Axioskop 2 Plus incident light fluorescence microscope (Zeiss) equipped with a CCD camera (Axiovision Cam, Zeiss), with a resolution of 1300 × 1030 pixels, configured for fluorescence microscopy, and interfaced to a computer workstation, provided with an interactive and automatic image analyser (Axiovision, Zeiss). Digital images were acquired using standard filters setup for Cy3, Alexa Fluor 488 or DAPI. At least 100 cells for each treatment were counted in order to quantified the incidence of Cytokeratin-8 and *α*-SMA positive oAEC.

For the AM immunofluorescence analysis was used Nikon A1r confocal microscope interfaced to a computer workstation, provided with NIS-Elements 4.4 software (for images acquisition) and with NIS-Elements Advanced Research imaging software (for post-processing analysis).

### Amniotic membrane immunohistochemistry analysis

Amniotic membrane pieces were collected and fixed in 4% paraformaldehyde for 48 hours, washed in dH2O, dehydrated and embedded with consecutive passages in dehyol 95%, dehyol 100%, xylene and hot paraffin, each step lasting one hour. Serial paraffin sections of 5 μm thickness were collected on poly-L-lysine-coated slides and sequentially processed for investigations. In detail, each sheet of amniotic membrane was subjected, at least in triplicate, to the following hematoxylin (Merk, Darmstadt, Germany)/eosin (Merk) staining to identify the epithelial layer of amniotic membrane and to analyse the morphological structure and the membrane integrity. Morphological analyses were performed with an Axioscop 2 Plus epifluorescence microscope (Zeiss, Oberkochen, Germany) equipped with a cooled color charge coupled device camera (CCD; Axiovision Cam, Zeiss) interfaced to a computer workstation and provided with an interactive and automatic image analyzer (Axiovision, Zeiss).

### Proliferation Assay

In order to evaluate the effects of P_4_ supplementation on oAEC proliferation and viability, the MTT assay (M5655-1G, Sigma) was performed. This method is based on the reduction of tetrazolium salt, MTT, to yellow formazan compounds, by mitochondrial dehydrogenase only in viable cells. CTR and 10, 25 or 50 *µ*M P_4_-treated oAEC were seeded into 96-well plates (0.3 × 10^5^ cells/well) until reaching 70% confluence. The blanks point was identified by the wells contained only culture medium (supplemented or not with P_4_). Afterwards, 20 *µ*L of MTT (5 mg/mL in PBS) was added in each well and the plates were incubated at 37 °C for 3.5 h. The formazan crystals were then dissolved in 100 *µ*L of DMSO. The absorbance (Abs) of the resulting solution was measured at 595 nm and for each sample were subtracted the relative blank absorbance. Finally, the percentage of proliferation was calculated as the absorbance of P_4_-treated cells divided for the absorbance of CTR cells and multiplied by 100. The net absorbance of CTR cells was taken as 100% of proliferation.

### Protein Extraction and Western Blotting

Total proteins were extracted from CTR and P_4_-treated oAEC at passage 3 in lysis buffer (50 mM Tris HCl pH 8, 150 mM NaCl, 1 mM EDTA, 100 mM NaF, 10% Glycerol, 1 mM MgCl_2_, 1% Triton X-100) with Phosphatase Inhibitor Cocktail (1:100) (P5726, Sigma) and Protease Inhibitor Cocktail (1:100) (P8340, Sigma). After five cycle of sonication of 30 seconds each, samples were put on ice for 15 minutes and then centrifuged at 14000 g for 15 minutes at 4 °C. Protein concentration was determined by using Thermo Scientific NanoDrop 2000c UV-Vis spectrophotometer and Quick Start™ Bradford 1x Dye Reagent (BioRad). Afterwards, 30 μg of total protein was separated onto Mini-PROTEAN^®^ Precast Gel (BioRad) and then transferred to nitrocellulose membranes (Millipore, Bedford, MA) at 30 V for 16 hours at 4 °C. Membranes were subsequently incubated with 5% notfat-dried milk (Sigma) in buffer containing 0.1% (v/v) Tween 20 in Tris-buffered saline (T-TBS) for 1 h, at 4 °C. Primary antibodies against p-Smad 2 (3108, Cell Signalling), Smad 2/3 (3102, Cell Signalling), and GAPDH (G9545, Sigma) were incubated overnight according manufacturer instructions. Finally, membranes were incubated with horseradish peroxidase–conjugated anti-Rabbit secondary antibody (31461, Pierce™ Antibody) for 1 h, at 4 °C and target proteins visualized by Westar ETA C 2.0 ECL substrate (XLS070,0250, Cyanagen) according manufactory instructions.

### oAEC basal genes expression

Real-time qPCR was performed in order to compare the mRNA expression of specific genes (Table 1) in CTR and P_4_-treated oAEC. In detail, total mRNA was extracted by using TRIzol (Sigma) according to the manufacturer instructions. Integrity and size distribution were evaluated by 1% agarose gel electrophoresis and GelRed staining (Biotium).

**Table 1:** Sequences of primers and conditions used in real-time qPCR.

Gene	Forward Sequence	Reverse Sequence	Annealing Temperature (°C)
*Vimentin*	5′-GACCAGCTCACCAACGACA-3′	5′-CTCCTCCTGCAACTTCTCCC-3′	65.2
*Snail*	5′-GTCGTGGGTGGAGAGCTTTG-3′	5′-TGCTGGAAAGTGAGCTCTGG-3′	66.4
*Twist*	5′-GCCGGAGACCTAGATGTCATTG-3′	5′-CCACGCCCTGTTTCTTTGAAT-3′	66.9
*Oct4*	5′-CTGCAGAAGTGGGTGGAGGAA-3′	5′-CTGCAGTGTGGGTTTCGGGCA-3′	68.7
*Sox2*	5′-CACCCGCATGTACAACATGAT-3′	5′-TCTTAGGATTCTCTTGGGCCA-3′	67.7
*Nanog*	5′-TGGATCTGCTTATTCAGGACAG-3′	5′-TGCTGGAGGCTGAGGTATTTC-3′	65.4
*IL-1β*	5′-ACGAACATGTCTTCCGTGAT-3′	5′-ACCAGGGATTTTTGCTCTCT-3′	62.1
*IL-6*	5′-ACCTGGACTTCCTCCAGAAC-3′	5′-TTGAGGACTGCATCTTCTCC-3′	62.0
*IL-12*	5′-TCAAACCAGACCCACCCAAG-3′	5′-CACAGATGCCCATTCACTCC-3′	65.6
*IL-10*	5′-CCAGGATGGTGACTCGACTAG-3′	5′-TGGCTCTGCTCTCCCAGAAC-3′	65.3
*IL-4*	5′-AAGCCCTCAGCTAAGCATGT-3′	5′-AGGCATCACAGGCTCAAGTC-3′	63.2
*TGF-β*	5′-GAAGTCTAGCTCGCACAGCA-3′	5′-CACGTGCTGCTCCACTTTTA-3′	64.0
*MMP-1*	5′-AAGATGTGGAGACGGTGCAG-3′	5′-CAGTCACTCTCAGCCCGAAG-3′	65.2
*MMP-2*	5′-ACATACAGGATCATTGGCTACACA-3′	5′-CGAAGGCATGAGCCAGGAG-3′	64.2
*MMP-13*	5′-GCCAGAACTTCCCAACCGTA-3′	5′-GTGAAGGGCTGCACTGATCT-3′	64.5
*GAPDH*	5′-TCGGAGTGAACGGATTTGGC-3′	5′-CCGTTCTCTGCCTTGACTGT-3′	64.4

Quantification of total mRNA samples was assessed by using Thermo Scientific NanoDrop 2000c UV-Vis spectrophotometer at 260 nm. Digestion of genomic DNA was carried out by DNaseI (Sigma) exposing the samples for 15 minutes at RT. cDNA was synthetized from 1 *µ*g of total RNA of each sample was used for reverse transcription reaction with Random Hexamers primer and Tetro Reverse Transcriptase (Bioline) at final volume of 20 *µ*L, according to the manufacturer instructions. Afterwards, Real-time qPCR analysis was performed by using SensiFAST^TM^ SYBR Lo-ROX kit (Bioline) by adjusting the manufacturer instruction to final volume of 15 *µ*L. The reaction was carry out with 7500 Fast Real-time PCR System (Life Technologies) by using the two-step cycling protocol for 40 cycles (10 second at 95 °C for denaturation and 30 second at 60 °C for annealing/extension) followed by melt-profile analysis (7500 Software v2.3). The relative expression level of mRNA was calculated by the ΔΔ*Ct* method. For details on primers sequences see Table 1.

### oAEC cytokines expression induced by LPS stimulation

The effect of LPS stimulation (1 *µ*g/mL for 24 hours) were analyses on oAEC at 1 and 3 passage by detecting cytokines expression. More in detail, the IL-10, the IL-4 and TGF-b were recorded on cultural medium (CM) using ELISA whereas *IL-10*, *IL-4*, and *TGF-*β were analyzed by using Real-Time qPCR.

Elisa was performed by using Nori Sheep IL-10, IL-4 and TGF-β ELISA Kits (Genorise Scientific, Inc). Standard or CM samples obtained by CTR and P_4_-treated cells were added to the microtiter plate wells with a horseradish peroxidase (HRP) conjugated antibodies and processed according manufacturer instructions. The optical density of each well was determined by using a microplate reader set to 450 nm and subtracting the corresponding reading at 540 nm for each well.

Real-Time qPCR analysis was performed in order to compare the immunomodulatory cytokines mRNA basal and LPS-induced expressions. The relative expression level of mRNA was calculated by the ΔΔ*Ct* method.

### Statistical Analysis

Data reported in this paper are the mean ( ± SEM) of at least 3 independent experiments, each performed in triplicate. All statistics were performed using Prism 6 (GraphPad). Two-way ANOVA’s were performed on data sets with two independent variables (stemness and EMT-related gene expression in CTR and P_4_-treated cells over passages) and one-way ANOVA’s with Tukey correction for multiple comparisons were performed on data sets with a single independent variable. Moreover, t-test was used to determine statistically significant differences in cytokine expression between CTR and P_4_-treated cells and in EMT-related gene expression between cells exposed to P_4_ at passage 4 and cells experienced P_4_-withdrawal. At least a p value < 0.05 was considered statistically significant.

### Data Availability

The datasets generated during and/or analysed during the current study are available from the corresponding author on reasonable request.

## Electronic supplementary material

Supplementary Information
