# Supplementary material for: Progesterone prevents epithelial-mesenchymal transition of ovine amniotic epithelial cells and enhances their immunomodulatory properties
Source: Sci Rep. 2017 Jun 19;7:3761. doi: 10.1038/s41598-017-03908-1 (PMC5476612; doi:10.1038/s41598-017-03908-1)
Supplement: Supplementary file 1 — Supplementary Information [file 41598_2017_3908_MOESM1_ESM.pdf]

Supplementary Information

Progesterone prevents epithelial-mesenchymal transition of ovine amniotic epithelial cells and enhances their immunomodulatory properties

Angelo Canciello[1], Valentina Russo[1], Paolo Berardinelli[1], Nicola Bernabò[1], Aurelio Muttini[1], Mauro Mattioli[2] & Barbara Barboni[1]

[1]Faculty of Bioscience and Technology for Food, Agriculture and Environment, University of Teramo, Via Renato Balzarini 1, 64100, Teramo, Italy. [2]Istituto Zooprofilattico Sperimentale dell'Abruzzo e del Molise (IZSAM) "G. Caporale", Campo Boario, 64100, Teramo, Italy. Correspondence and requests for materials should be addressed to A.C. (email: acanciello@unite.it)

*Corresponding author.
Address correspondence to Angelo Canciello, Faculty of Bioscience and Technology for Food, Agriculture and Environment, University of Teramo, Via Renato Balzarini 1, 64100, Teramo, Italy.

E-mail address: acanciello@unite.it or angelocanciello@yahoo.it

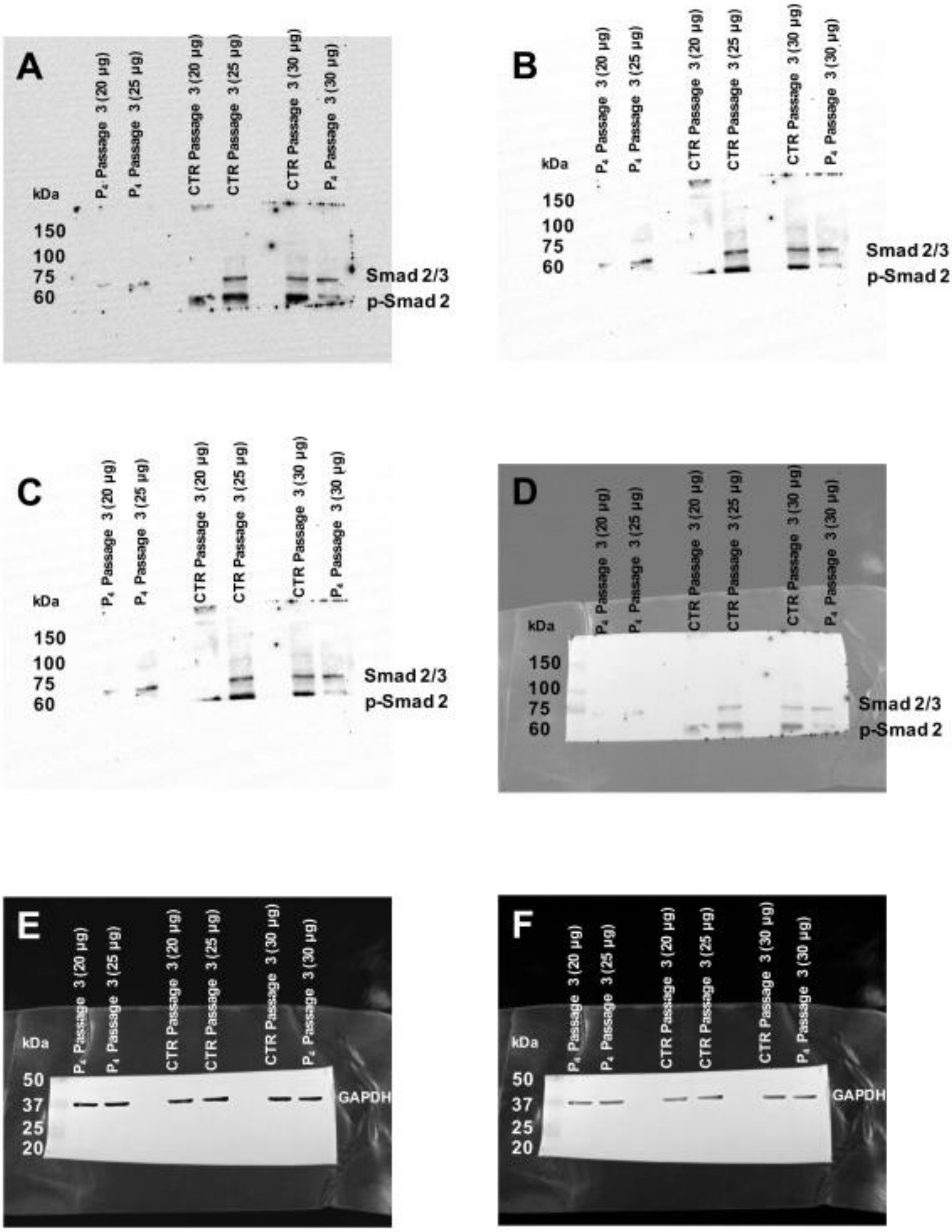

Supp.Fig1. Full-length blots for the indicated figures from the main text. (A-C) Multiple exposures of blots for p-Smad 2 and Smad 2/3. **(D)** Merged image between membrane and blots for p-Smad 2 and Smad 2/3. **(E-F)** Merged image between membrane and blots for GAPDH. The antibodies used in our experiment was previously published in the study titled "*Galectin-3 Regulates Atrial Fibrillation Remodeling and Predicts Catheter Ablation Outcomes*" by Takemoto Y. et al., 2016 (doi:10.1016/j.jacbts.2016.03.003).
